# 1909. Steak with a Side of Omicron? Efficacy of Coordinated Outbreak Response during an early COVID-19 Omicron Outbreak amongst Restaurant Workers in Riverside County, CA

**DOI:** 10.1093/ofid/ofac492.1536

**Published:** 2022-12-15

**Authors:** Shunling Tsang, Wendy Hetherington, Wendy Hetherington, Juan Landeros-Tavera, Jennifer Chevinsky, Marshare Penny

**Affiliations:** Riverside University Health System Public Health, Riverside, California; Riverside University Health System Public Health, Riverside, California; Riverside University Health System Public Health, Riverside, California; Riverside University Health System Public Health, Riverside, California; Riverside University Health System Public Health, Riverside, California; Riverside University Health System Public Health, Riverside, California

## Abstract

**Background:**

On Dec 9, 2021, Riverside University Health System-Public Health (RUHSPH) identified a large outbreak of COVID-19 with staff at an upscale restaurant. Omicron was emerging as a Variant of Concern (VOC) and not well understood within the county. This study offers safe reopening strategies during a VOC outbreak.

**Methods:**

RUHSPH worked with county supervisors, counsel, environmental health, and California Department of Public Health outbreak consultation team. Collaboration with corporate restaurant leaders resulted in voluntary closure of operations on Dec 21, 2021. RUHSPH teams were deployed to test and interview 33 staff over 2 days. Specimens were sent to RUHSPH lab for rapid processing. Positive samples were sent to California Viral and Rickettsial Disease Laboratory for whole genomic sequencing (WGS). Outbreak mitigation measures were agreed upon with restaurant leaders. Demographics of cases were summarized using descriptive statistics. Differences by vaccination status and Omicron VOC confirmation were tested with Pearson's Chi-Square.

**Results:**

Between Nov 18-Dec 28, 2021, 31 cases were identified with a mean age of 34.9 years, including 4 positives during the 2-day outbreak investigation. (**Table 1**) Staff were demographically diverse with no significant differences among those who completed their primary vaccine series. 7 Omicron BA.1 cases were detected amongst staff who completed their primary vaccine series. (**Figure 1**) Significantly more Omicron cases were observed among females (p< 0.05). Phylogenetic analysis of the Omicron specimens showed three distinct introductions with one clear cluster of 4 cases.

A framework of staff clearance was detailed with restaurant leaders. Clearance criteria allowed for reopening the next day. Contact tracing occurred for 79 total staff. No additional cases were identified amongst restaurant staff and the outbreak was closed on Jan 2, 2022.

**Table 1.**
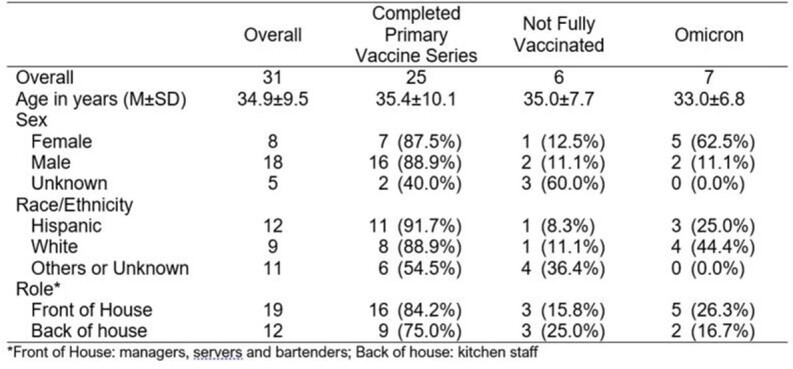
Characteristics of the 31 employees that tested positive, including those tested during the 2-day investigation.

**Figure 1.**
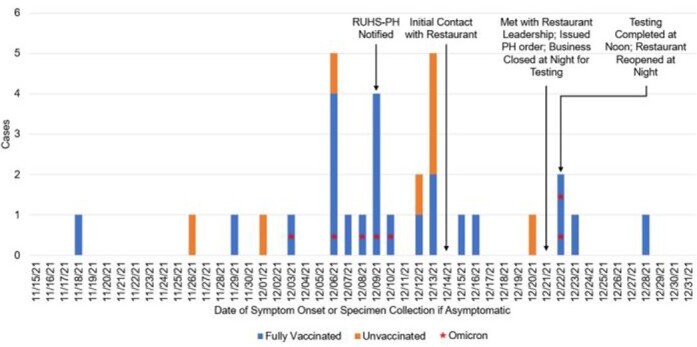
Employees Infected with COVID-19 by Date of Symptom Onset or Specimen Collection

**Conclusion:**

This response highlights the importance of collaboration between state, local, and workplaces to develop a concise approach in an emerging VOC outbreak. WGS provided evidence of distinct introductions and workplace transmission amongst restaurant staff. This approach will be imperative to evaluate and contain future VOC outbreaks.

**Disclosures:**

**All Authors**: No reported disclosures.

